# Supplementary material for: Multivariate adaptive shrinkage improves cross-population transcriptome prediction and association studies in underrepresented populations
Source: HGG Adv. 2023 Jul 1;4(4):100216. doi: 10.1016/j.xhgg.2023.100216 (PMC10589725; doi:10.1016/j.xhgg.2023.100216)
Supplement: Document S1. Figures S1–S3 [file mmc1.pdf]

HGGA, Volume *4*

Supplemental information

Multivariate adaptive shrinkage improves cross-population transcriptome prediction and association studies in underrepresented populations

Daniel S. Araujo, Chris Nguyen, Xiaowei Hu, Anna V. Mikhaylova, Chris Gignoux, Kristin Ardlie, Kent D. Taylor, Peter Durda, Yongmei Liu, George Papanicolaou, Michael H. Cho, Stephen S. Rich, Jerome I. Rotter, NHLBI TOPMed Consortium, Hae Kyung Im, Ani Manichaikul, and Heather E. Wheeler

1. Supplemental Figures

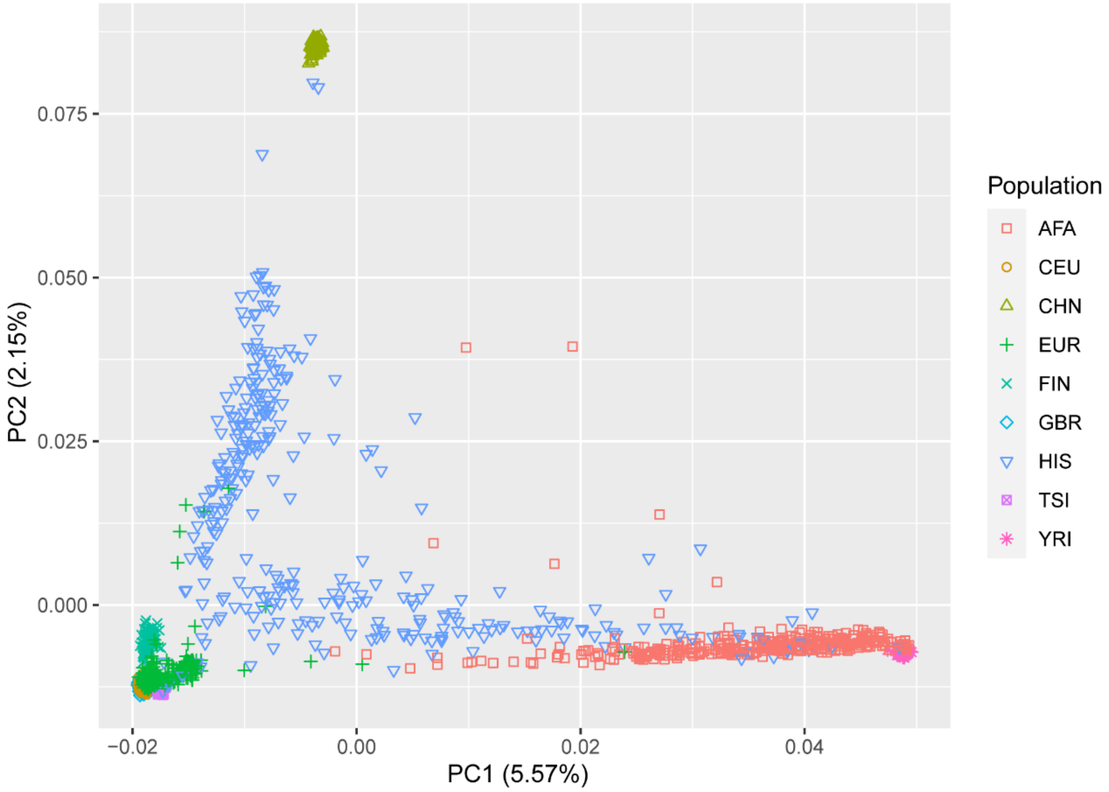

Figure S1: Genotype principal component analysis. Plot of the first two principal components of TOPMed MESA populations with Geuvadis populations. Proportions of variance explained by the first two principal components estimated using the top 1670 principal components. AFA = African American (TOPMed), CEU = Utah residents with Northern and Western European ancestry (Geuvadis), CHN = Chinese (TOPMed), EUR = European (TOPMed), FIN = Finnish in Finland (Geuvadis), GBR = British in England and Scotland (Geuvadis), HIS = Hispanic/Latino (TOPMed), TSI = Toscani in Italy (Geuvadis), YRI = Yoruba in Ibadan, Nigeria (Geuvadis).

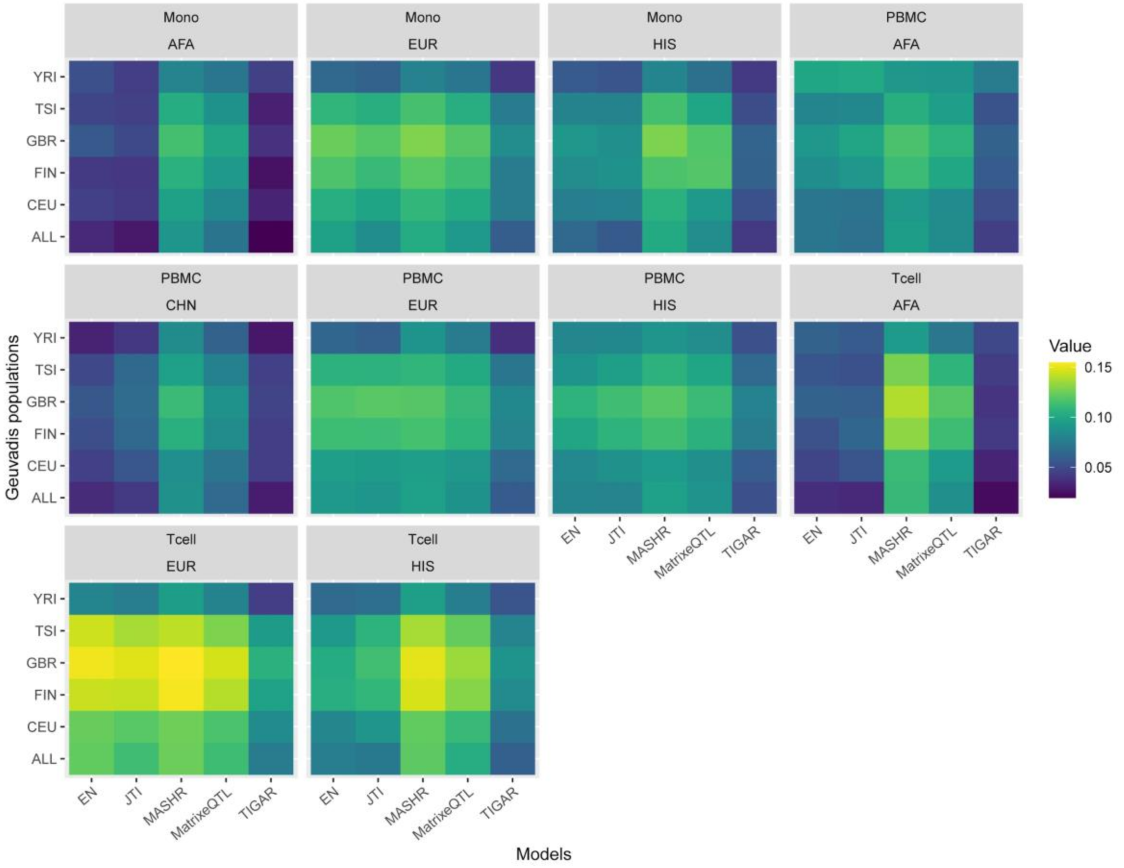

Figure S2: Overall prediction performance of MESA population models in Geuvadis. Prediction performance (median Spearman's rho) of EN, JTI, MASHR, MatrixeQTL, and TIGAR MESA population models in all Geuvadis populations.

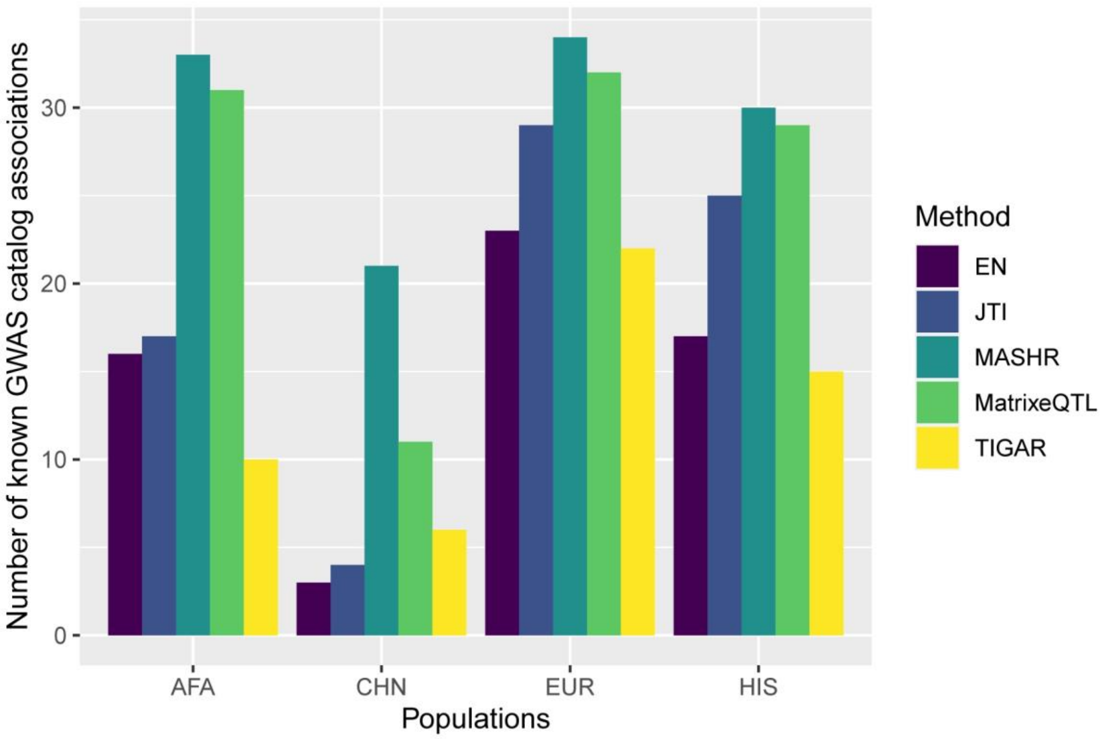

Figure S3: Number of significant S-PrediXcan gene-trait pairs in PAGE and PanUKBB GWAS summary statistics that have been reported in the GWAS catalog. Total number of significant gene-trait pairs discovered by each MESA population model (considering the union of the three tissues), by method.

2. Supplemental Tables

Table S1: PAGE and PanUKBB summary statistics used in this study.

Table S2: Performance comparisons of PBMC AFA and EUR MESA transcriptome prediction models in the GBR and YRI Geuvadis populations between all methods.

Table S3: Pairwise comparisons of the performance of EN, JTI, MASHR, MatrixeQTL, and TIGAR MESA transcriptome prediction models in all Geuvadis populations.

Table S4: Compiled S-PrediXcan gene-trait pair discoveries, significant in PAGE and PanUKBB GWAS summary statistics with the same direction of effect.

Table S5: List of NHLBI TOPMed consortium members.
